# Munduruku Indigenous children: health situation in an area with high mercury exposure

**DOI:** 10.11606/s1518-8787.2025059006403

**Published:** 2025-06-11

**Authors:** Cristina Barroso Hofer, André Reynaldo Santos Périssé, Ana Cláudia Santiago de Vasconcellos, Paulo Victor de Sousa Viana, Joseph W. Kempton, Marcelo de Oliveira Lima, Iracina Maura de Jesus, Sandra de Souza Hacon, Paulo Cesar Basta

**Affiliations:** I Universidade Federal do Rio de Janeiro Faculdade de Medicina Instituto de Pediatria e Puericultura Martagão Gesteira Rio de Janeiro RJ Brazil Universidade Federal do Rio de Janeiro. Faculdade de Medicina. Instituto de Pediatria e Puericultura Martagão Gesteira. Rio de Janeiro, RJ, Brazil; II Fundação Oswaldo Cruz Escola Nacional de Saúde Pública Sergio Arouca Departamento de Endemias Samuel Pessoa Rio de Janeiro RJ Brazil Fundação Oswaldo Cruz. Escola Nacional de Saúde Pública Sergio Arouca. Departamento de Endemias Samuel Pessoa. Rio de Janeiro, RJ, Brazil; III Fundação Oswaldo Cruz Escola Politécnica de Saúde Joaquim Venâncio Laboratório de Educação Profissional em Vigilância em Saúde Rio de Janeiro RJ Brazil Fundação Oswaldo Cruz. Escola Politécnica de Saúde Joaquim Venâncio. Laboratório de Educação Profissional em Vigilância em Saúde. Rio de Janeiro, RJ, Brazil; IV Fundação Oswaldo Cruz Escola Nacional de Saúde Pública Sergio Arouca Centro de Referência Professor Hélio Fraga Rio de Janeiro RJ Brazil Fundação Oswaldo Cruz. Escola Nacional de Saúde Pública Sergio Arouca. Centro de Referência Professor Hélio Fraga. Rio de Janeiro, RJ, Brazil; V Imperial College London Faculty of Medicine Medical School Building London United Kingdom Imperial College London. Faculty of Medicine. Medical School Building, St Mary’s Hospital. London, United Kingdom; VI Ministério da Saúde Secretaria de Vigilância em Saúde e Ambiente Instituto Evandro Chagas Ananindeua PA Brazil Ministério da Saúde. Secretaria de Vigilância em Saúde e Ambiente. Instituto Evandro Chagas. Ananindeua, PA, Brazil

**Keywords:** Mercury Poisoning, Environmental Health, Indigenous People, Amazonian Ecosystem

## Abstract

**OBJECTIVE:**

The Munduruku indigenous people are among those most severely impacted by mercury (Hg) contamination in the Amazon region. Mercury exposure can have severe consequences for the physical and cognitive development of children. We aimed to describe the health assessment in indigenous Munduruku children in the Tapajós River basin, possibly exposed chronically to Hg.

**METHODS:**

A cross-sectional study with children <12 years old living in the *Sawré Muybu* (SM), *Poxo Muybu* (PM), and *Sawré Aboy* (SA) villages. We collected data between October 29^th^ and November 9^th^, 2019, through interviews and clinical evaluation (child neurodevelopment tests and anthropometric measurements), and measured blood hemoglobin levels and Hg in hair samples. The history of immunization and other health issues were collected from the Brazilian health booklets. We investigated the prevalence of Hg exposure ≥ 6.0 µg/g. The measure of association used was the Prevalence Ratio (PR), with a 95% CI.

**RESULTS:**

We examined 83 children, 40 in SM, 15 in SA, and 28 in PM. 51.8% were girls, 45.7% had completed the vaccination schedule, 16.0% had changes in the neurodevelopment test, and 13.9% had anemia. Their weight and height/length averages were (Z-scores) -0.86 and -1.59, respectively. 45.1% of children had Hg-levels ≥ 6.0 µg/g. The prevalence of mercury contamination in children in SA was almost four times greater (PR:3.66; 95%CI 2.17-6.18) than that in children in SM, and girls were almost twice as likely (PR:1.67; 95%CI 1.07-2.62) to have mercury levels ≥ 6.0 µg/g, when compared with boys in the study villages.

**CONCLUSIONS:**

The higher concentration of hair Hg-levels occurred in SA, where Hg exposure is higher. Although we cannot prove causality, we believe that understanding the possible health impacts of mercury exposure among the Munduruku children is vital for developing strategies to mitigate these effects and for supporting the fight for their rights and the protection of their territories.

## INTRODUCTION

Mercury exposure is a significant environmental and public health concern affecting many traditional populations in the Amazon Basin, particularly indigenous communities located near artisanal small-scale gold mining (ASGM) activities^1^. Currently, the Munduruku people, an indigenous group residing in the Tapajós River region of the Brazilian Amazon, are among those most severely impacted by mercury contamination^4^. The continued use of mercury in mining has led to extensive contamination of water bodies, fish, and other local food sources, which are crucial to the traditional lifestyle of the Munduruku people. This chronic exposure presents serious health risks, especially for vulnerable populations such as children, who are particularly susceptible to mercury’s harmful effects^5^.

In children, mercury exposure can have severe consequences for their physical and cognitive development. Methylmercury, the most toxic form of mercury, can cross the placental barrier, affecting fetal development even before birth^5^. A systematic review of 34 studies conducted in the Amazon region revealed that the mercury body burden in children was 2–10 times higher than the recommended levels. The neurological outcomes included in this study were cognitive, visual, motor, somatosensory, and emotional deficits^6^. Despite evidence that continued exposure to contaminated fish increases the likelihood of neurodevelopmental issues, behavioral problems, and other cognitive impairments, there remains a need for comprehensive data and targeted public health interventions^8^.

The middle Tapajós River region, where numerous Munduruku communities are located, has become a focal point for studying the effects of mercury exposure. Recent research has not only identified elevated mercury levels that exceed international health guidelines but also linked these levels to neurological^9^ and psychosocial disorders^10^ in adults, as well as methylmercury contamination in fish^11^ within three specific communities. There is still a lack of understanding regarding the full impact of mercury exposure on the growth, development, and overall well-being of Munduruku children, as well as the broader socio-cultural consequences of this environmental health crisis^10^.

Even though Brazilian federal law prohibits mining activities on Indigenous Lands (IL), the extent of mining within these areas in the Brazilian Legal Amazon has dramatically increased. The total mining area expanded from 7.45 km^2^ in 1985 to 102.16 km^2^ in 2020, representing a growth of 1,271%. This activity is heavily concentrated, with 95.0% of the mining occurring within just three IL: Munduruku, Kayapó, and Yanomami^12^.

Our study aimed to describe the health situation among indigenous children of the Munduruku ethnic group chronically exposed to mercury in three communities located in the region of the middle Tapajós River in the state of Pará, Brazilian Amazon.

## METHODS

### Study Design and Population

We conducted a cross-sectional study in the *Sawré Muybu* IL from October 29^th^ to November 9^th^, 2019, during the dry season. The IL, traditionally occupied by the Munduruku people, is located in the municipalities of Itaituba and Trairão, Pará^4^.

The Tapajós River basin is inhabited by different groups of indigenous people. The Munduruku live in the Munduruku IL officially recognized by the Federal Government^13^. However, they also live in the Sawré Muybu IL, which, although traditionally occupied by the Munduruku people, is not yet recognised by the public authorities. In general, these are areas where the structure of houses is marked by the unavailability of drinking water for human consumption, the absence of bathrooms for the exclusive use of families and inappropriate waste management, revealing a significant sanitation deficit^4^. The Rio Tapajós Special Indigenous Health District (DSEI) is the public sector responsible for the health of the indigenous people in the Tapajós River area and is located in the municipality of Itaituba. The DSEI provides health care to almost 15,000 indigenous people from nine different ethnic groups distributed amongst 173 villages, the majority of which are of the Munduruku ethnic group^14^.

We included people living in the *Sawré Muybu*, *Poxo Muybu* and *Sawré Aboy* villages, following a request by the *Pariri* Indigenous Association, which represents the Munduruku People of the Middle-Tapajós River. Basta et al., 2021, presented more information about the territories and characteristics of its populations^4^. In the analyzed villages, a population census was conducted. All resident children under the age of 12 years were invited to participate in the study. Therefore, probabilistic sampling methods were not used to select participants.

### Fieldwork

The study was carried out during the dry season in the Amazon, a period that, despite high temperatures and humidity, facilitates travel along roads and rivers in the region. All the material to be used in the research was taken to the villages in their own boats, driven by trained indigenous people. Travel between villages was also carried out in boats, while accommodation was guaranteed by the indigenous people in areas of the villages designated for the placement of hammocks. The proximity between the villages *Sawré Muybu* (Tapajós River) and *Sawré Aboy* (Jamanxin River) made field logistics in these two areas less difficult. Due to the distance and difficulties in traveling along the river in times of drought, the village of *Poxo Muybu* was the last one visited by the research team.

Data were collected through home visits and interviews with participating families, clinical and laboratory evaluation, collection of hair samples (used as Hg exposure biomarker), and review of children’s health booklets.

Home visits and interviews with families were carried out by a team composed of an indigenous anthropologist, a biologist, a nurse and a psychologist who were previously trained in the protocol. The interviewers used a questionnaire specially designed for this study based on the previous experiences of the research group^15,16^. The questionnaire was applied with the help of indigenous health agents (AIS), who work within the communities, and local indigenous leaders, including chiefs and teachers. They were structured with closed questions and the responses were recorded on electronic forms with the aid of tablets.

After home visits and interviews, the families were invited to participate in a clinical and laboratory evaluation, carried out by physicians (pediatrician, infectious diseases specialist and neurologist) in a location previously agreed with the community leaders.

### Health Situation Assessment

Indigenous children under the age of 12 from all villages had their health booklets reviewed for an analysis of vaccination coverage. Based on the data recorded in the child’s health booklet, it was possible to check the vaccine records, with dates and corresponding doses, and to compare the records with the current national immunization program schedule for the population studied. All missing records were reported to the nurse responsible for the multidisciplinary indigenous health team (EMSI).

The children were weighted and measured twice, and the average was used as the final measure. The mean of weight and height/length were recorded in the Child Growth and Development Program by the EMSI, and for children up to 59 months of age were transformed into Z-scores (adjusted for sex and age), and the weight/age (W/A), height/age (H/A), and body mass/age (BMI/A) indices were calculated using a reference population according to the World Health Organization^17^.

A HemoCue^®^ device model HB 301-System was used to measure the capillary hemoglobin. The normal cut-off for no anaemia was according to the age group of the participants: i) children aged 6–23 months: ≥ 10.5 g/dL; ii) children aged 24 to 59 months: ≥ 11.0 g/dL; iii) children from 5 to 11 years old: ≥ 11.5 g/dL^18^.

A neurodevelopmental assessment was performed by using the Denver II screening test in children aged 0 to 6 years without morphological changes. The Denver II test, composed of 125 items divided into personal-social, fine motor skills, language and broad motor skills areas, assesses and identifies children at risk for developmental delay, but it was not developed to diagnose learning or emotional disorders^19^. We were not able to find any study aimed at validating a test for neurodevelopment assessment in the indigenous population in Brazil.

### Collection of Human Samples (Hair)

Samples of hair from the occipital region were collected from all participants and removed close to the scalp with the aid of stainless steel dissection scissors. The samples were stored in paper envelopes, individually identified, and sent for analysis of total mercury levels (HgT) in the Toxicology Laboratory, Environment Section of the Evandro Chagas Institute (IEC). We considered the level of ≥ 6.0 µg/g of mercury in hair samples as a health risk indicator, following the parameters of previous studies carried out in the Amazon region^3,20^. A detailed description of the determination of the mercury levels in the samples can be accessed elsewhere^4^.

### Statistical Analysis

The level of mercury in the hair (outcome) was categorized into < 6.0 vs ≥ 6.0 µg/g. The prevalence of mercury contamination was estimated by the ratio between the number of children under 12 years of age with mercury levels ≥ 6.0 µg/g and the child population at the same age in each of the three villages.

Both vaccination coverage and neurodevelopment assessment using the Denver II test were categorized as “age-appropriate” and “not age-appropriate”. Vaccine coverage was “age-appropriate” when all vaccines recommended for the age had been registered on the card, and “not age-appropriate” for children with at least one vaccine recommended for their age not registered on the card.

Anthropometric data for the first 60 months of life were described based on the World Health Organization graphics from 2006^17^.

Pearson’s chi-square test was used to study the association between the categorical variable and the dichotomous outcome. To estimate the association between the outcome and the variables under study, we used a logistic regression model with a conditional prevalence ratio (PR). Variables with p < 0.20 in the bivariate analysis and those likely to act as confounding variables were selected and included in the multivariate model. The variables that presented a 5% significance level (p < 0.05) remained in the final model. The 95% confidence intervals were obtained using the delta method and cluster bootstrap. For all statistical tests, a significance level of 5% (p < 0.05) was considered.

We used the statistical software R version 4.0.5 (R Core Team, 2021) for data analysis.

### Ethical Aspects

The study protocol was evaluated by the Research Ethics Committee of the Oswaldo Cruz Foundation National School of Public Health (CEP/ENSP) and the National Research Ethics Committee (Conep) (CAAE: 65671517.1.0000.5240). Written informed consent was obtained from local leaders after discussion with the communities about the project and clarification of eventual concerns. All interviews and study procedures were performed with the help of a Munduruku translator.

The research was requested by the community, and we followed the guidelines of Convention 169 of the International Labor Organization (ILO), implementing a prior community consultation and a preliminary presentation of the research protocol in August 2019, before the start of the fieldwork. At the time, the study objectives were presented and discussed. Interviews and data collection started only after the participants had their questions answered and were given formal consent in the Informed Consent Form by the children’s guardians.

## RESULTS

In total, 83 out of 85 (97.6%) children under 12 years old were included and evaluated, 40 (48.2%) residing in *Sawré Muybu*, 28 (33.7%) in *Poxo Muybu* and 15 (18.1%) in *Sawré Aboy* (Table 1). Forty-three girls (51.8%) and 40 boys (48.2%) were examined. There were 16 (19.3%) children under one year old, 24 (28.9%) between 1 and 5 years old and 43 (51.8%) greater than or equal to 5 years old. The health booklets of 81 children were reviewed, and only 37 (45.7%) had a complete vaccination schedule (according to their age). Pentavalent (difteria, tetanus, pertussis, hepatitis B and type B *H. influenzae* vaccine)(18.5%), conjugated Pneumococcal 10-valent vaccine (16.1%), and conjugate Menigococcal C vaccine (22.2%) were the most absent immunizations. The lowest vaccination coverage was found in the *Sawré Aboy* village, where only 4 of 14 children (28.6%) had a complete vaccination schedule for their age.

**Table 1:** Demographical and clinical characteristics, according to the villages of residence, Munduruku indigenous children. State of Pará, Brazil, 2019.

Characteristics	Categories	Sawré Muybu (n = 40; 48.2%)	Poxo Muybu (n = 28; 33.7%)	Sawré Aboy (n = 15; 18.1%)	Total (n = 83; 100%)
Sex	Female	22 (55.0)	16 (57.1)	5 (33.3)	43 (51.8)
	Male	18 (45.0)	12 (42.9)	10 (66.7)	40 (48.2)
Age group in years	≤ 1	6 (15.0)	5 (17.9)	5 (33.3)	16 (19.3)
	1–5	14 (35.0)	6 (21.4)	4 (26.7)	24 (28.9)
	≥ 5	20 (50.0)	17 (60.7)	6 (40.0)	43 (51.8)
Timeliness and coverage of vaccination (n = 81)	Yes	17 (42.5)	16 (59.3)	4 (28.6)	37 (45.7)
	No	23 (57.5)	11 (40.7)	10 (71.4)	44 (54.3)
Appropriate Denver II test (n = 56)	Yes	22 (81.5)	18 (94.7)	7 (70.0)	47 (84.0)
	No	5 (18.5)	1 (5.3)	3 (30.0)	9 (16.0)
Anaemia (according to age) (n = 79)	Yes	4 (10.3)	5 (18.5)	2 (15.4)	11 (13.9)
	No	35 (89.7)	22 (81.5)	11 (84.6)	68 (86.1)
Mercury level (n = 82)	0–2 µg/g	4 (10.2)	1 (3.6)	0 (0.0)	5 (6.0)
	> 2–4 µg/g	13 (33.3)	5 (17.8)	1 (6.7)	19 (23.2)
	> 4–6 µg/g	10 (25.7)	9 (32.2)	2 (13.3)	21 (25.7)
	≥ 6 µg/g	12 (30.8)	13 (46.4)	12 (80.0)	37 (45.1)

Nine (16.0%) of the 56 children under 6 years of age eligible for the Denver II test presented with abnormalities. It is worth mentioning that in the *Sawré Aboy* village, 3 in 10 children had problems related to neurodevelopment (Table 1). In total, six of the children assessed had limitations in the language domain.

Eleven children (13.9%) in all villages had anemia according to their age. Only five children (6.0%) presented a normal level of mercury in their hair according to the WHO criteria (0–2 µg/g). The prevalence of elevated hair concentrations of mercury (≥ 6 µg/g) reached 30.8% in *Sawré Muybu*, 46.4% in *Poxo Muybu*, and 80.0% in *Sawré Aboy* (Table 1). It is worth noting that the highest level of contamination by mercury (22.1 µg/g) was registered in a 5-year-old female child.

The Munduruku boys and girls have weights and statures slightly below the global average (according to WHO growth curves). Their average weight (z-score) was -0.86 (ranging from -3.16 to 1.71) and their average stature (z-score) was -1,59 (ranging from -3.63 to 0.6).

Thirty-seven children (45.1%) presented with mercury levels equal to or above 6.0 µg/g. The average level of mercury in children was 6.9 (± 4.8) μg/g, the median was 5.5 μg/g, and the values ranged between 1.4 and 22.1 μg/g. Girls were more affected than boys (59.0% x 41.0%) and contamination was more evident in those older than 5 years (59.5%) (Table 2).

**Table 2:** Demographical and clinical characteristics, according to the mercury levels of contamination, in the Munduruku indigenous children. State of Pará, Brazil, 2019.

Characteristics	Categories	< 6.0 µg/g (n = 45; 54.9%)	≥ 6.0 µg/g (n = 37; 45.1%)	Total (n = 82; 100%)	p-value^a^
Sex	Female	20 (44.0)	22 (59.0)	42 (51.0)	0.17
	Male	25 (56.0)	15 (41.0)	40 (49.0)	
Age group in years	≤ 1	12 (26.7)	3 (8.1)	15 (18.3)	0.09
	1–5	12 (26.7)	12 (32.4)	24 (29.3)	
	≥ 5	21 (46.6)	22 (59.5)	43 (52.4)	
Villages	*Sawré Muybu*	27 (60.0)	12 (32.4)	39 (48.0)	0.005
	*Poxo Muybu*	15 (33.3)	13 (35.2)	28 (34.0)	
	*Sawré Aboy*	3 (6.7)	12 (32.4)	15 (18.0)	
Timeliness and coverage of vaccination (n = 81)	Yes	17 (37.8)	20 (57.0)	37 (46.0)	0.08
	No	28 (62.2)	15 (43.0)	43 (54.0)	
Anaemia (according to age) (n = 78)	Yes	6 (14.6)	4 (10.8)	10 (12.8)	0.61
	No	35 (85.4)	33 (89.2)	68 (87.2)	

^a^ Pearson’s Chi-squared test.

The multiple logistic regression model revealed that the chances of mercury contamination in children in *Sawré Aboy* were almost four times greater (PR: 3.66; CI95% 2.17–6.18) than for children in *Sawré Muybu* and that girls were almost twice as likely (PR: 1.67; CI95% 1.07–2.62) to have mercury levels ≥ 6.0 µg/g, when compared with boys in the study villages (Table 3).

**Table 3:** Logistic regression model to elucidate associated factors with mercury levels of contamination (≥ 6,0µg/g-1), Munduruku indigenous children. State of Pará, Brazil, 2019.

Characteristics	Categories	Crude PR (95%CI)	p-value	Adjusted PR (95% CI)	p-value
Villages	*Sawré Aboy*	2.60 (1.52–4.45)	< 0.001	3.66 (2.17–6.18)	< 0.001
	*Poxo Muybu*	1.50 (0.81–2.80)	0.194	1.56 (0.87–2.79)	0.135
	*Sawré Muybu* (Ref.)	1.0			
Sex	Female	1.39 (0.85–2.30)	0.187	1.67 (1.07–2.62)	0.025
	Male (Ref.)	1.0			
Age group in years	≤ 1	0.39 (0.13–1.12)	0.082	-	
	1–5	0.97 (0.59–1.61)	0.928		-
	≥ 5 (Ref.)	1.0		-	
				
Anaemia (according to age) (n = 78)	Yes	0.82 (0.37–1.84)	0.637	-	-
	No (Ref.)	1.0			

PR: prevalence ratio; CI: confidence interval; Ref.: category of reference.

## DISCUSSION

Although studies investigating the effects of chronic exposure to mercury on the health of children living in Brazil have been expanding in recent years^16,21^, little is known about the impact among indigenous peoples. Herein, we carried out a situational health assessment, including levels of exposure to mercury, of 83 indigenous children of the Munduruku people from the region of the middle Tapajós River.

Our findings show that almost all children investigated were chronically exposed to mercury, and levels ≥ 6.0 µg/g were associated with the village of residence. However, the situation was even more worrying for children living in the *Sawré Aboy* village, where we found a high prevalence of mercury contamination together with low vaccination coverage and inadequate nutritional status. As reported by other authors, indigenous children in Brazil are more prone to anemia^22^, malnutrition^23^ and respiratory infections^24,25^. This combination results in high mortality rates due to preventable causes that can be tackled effectively by primary care^26^. The results presented show a concerning trend among Munduruku children, and for the children of *Sawré Aboy* in particular, and is in keeping with findings in other indigenous communities in the Amazon^3,15^. The *Sawré Aboy* village is located on the banks of the Jamanxim River, one of the main tributaries on the right bank of the Tapajós River, which is one of the areas currently most impacted by illegal mining. It is not surprising that children living in that region have the highest levels of Hg detected in their hair, indicating mercury contamination and other impacts of the presence of illegal mining.

Vaccine opportunity and coverage is representative of the quality of public health measures in a pediatric population, especially in times of pandemic, and a main reason to be described in a pediatric health assessment. Our results showed that less than half of the children investigated had a complete vaccination schedule for their age, with the worst vaccination coverage observed in the *Sawré Aboy* village (less than a third). Although there is a paucity of literature on vaccination coverage among indigenous peoples in Brazil, the consequences of low vaccination coverage on the spread of vaccine-preventable diseases are widely known^27^.

The study of the weight to age (W/A) curve over the first five years of life showed Munduruku children to have weights and statures slightly below the global average^17^. Considering that the stature average is lower than the weight average, the chronic nature of the deficits in nutritional status could be one of the explanations for our results. Malnutrition in the Brazilian indigenous population is a chronic problem that has been long reported. However, it remains current and challenging, being one of the main causes of morbidity and mortality for these groups in Brazil^26,28^.

All children investigated had some level of mercury in the hair samples analyzed. The average levels of mercury in children reached almost 7.0 μg/g, girls were more affected than boys, and contamination was more evident in those older than 5 years. The prevalence of contamination according to the villages showed an effect gradient, with approximately three, five and eight out of every 10 children evaluated showing mercury levels above 6.0 µg/g in *Sawré Muybu, Poxo Muybu* and *Sawré Aboy* villages, respectively. In our results, children living in the *Sawré Aboy* village and girls were more likely to present with mercury levels above our defined cut-off, in line with another study with the Munduruku people that revealed an increment of mercury concentration with age and higher levels among women^1^. This could reflect the cultural characteristics of the Munduruku people and may indicate that these girls could carry this background exposure to adulthood, amplifying the risk during the childbearing age.

Among children under 6 years who underwent the Denver II test, almost a fifth (16.0%) showed changes in neurodevelopment. The median hair mercury levels were not higher among the children who did not pass the Denver II test, as compared to those who passed. Considering the cross-sectional study design, we were not able to infer any relation between the test result and the hair mercury level; unmeasured prior mercury levels and a small number of children who performed the test jeopardized any further analysis. The changes described here for the language component of the Denver II test are in line with findings from other studies that analyzed non-indigenous Brazilian children. Vejrup et al. found in the Norwegian Mother and Child Cohort Study that levels of prenatal mercury exposure were inversely associated with language and communication skills at five years^29^. On the other hand, Dack et al., 2022, after reviewing 32 prospective studies that used 23 different scales (most commonly the Bayley Scales of Infant and Toddler Development) to evaluate neurodevelopmental performance, concluded that in most cases, the evidence for an association between mercury and neurodevelopment was weak^30^.

Other indigenous groups in the Amazon have also faced problems related to mercury contamination for at least two decades. High levels of contamination have been reported among the Yanomami of Roraima and at the *Sai Cinza* Indigenous Land, in the upper Tapajós River, where the authors had already recorded high levels of mercury contamination in the Munduruku population, both in children, in women of childbearing age and in adult men^1,3^.

Apart from the difficulties in data collection (field logistics) and problems with the study design (cross-sectional), other possible limitations should be pointed out. Our study covered only three villages in the region of the middle Tapajós River, including a limited number of 83 children under the age of 12. Despite being able to interview almost all children in the villages, the sample may not represent the entire population of the Munduruku children. The small number of children may also have diminished our ability to find associations between our exploratory variables and the presence of elevated hair Hg concentration. The complex interactions amongst different socioeconomic and environmental variables in the health-disease process of mercury contamination and the cross-sectional study design did not allow us to make any inference of causality in our study. Another potential limitation is the absence of specific instruments for collecting data on the relationship between health and the environment for ethnically different populations, especially those aimed at assessing child neurodevelopment. In the absence of any specific neurodevelopment test for the native Brazilian populations, we used Denver II because it is the most suitable for the proposed use. It is difficult to estimate the possible direction of the bias resulting from its use in the Munduruku children since it has not been validated for them. Therefore, such tests may not be entirely suitable for these populations, and future studies should evaluate other tests. Finally, due to linguistic restrictions, the interviews could have been more comprehensive, meaning some detailed information about local eating habits or other determinants that may be related to chronic exposure to mercury may have been missed. It follows that more in-depth studies are needed, with a longitudinal approach, including women of childbearing, pregnant women and children from birth to school age. Our research group is currently helping the local health authorities and the Munduruku people to conduct a cohort study to further explore the health-related problems associated with mercury exposure.

The study seeks to document the levels of exposure and the associated health outcomes among children, providing valuable data to inform public health policies and support community-driven interventions. Despite the study’s inherent limitations, we showed that chronic exposure to mercury constitutes a serious health threat to the Munduruku children. The high concentration of hair Hg-levels in the studied villages is compounded by the equally serious and worrying occurrence of malnutrition and anemia as well as structural problems such as the instability in the provision of public health services, education and sanitation services. These findings sit in the wider context of the permanent threat of invasion of traditional territory by miners, loggers and land grabbers interested in the riches of the Amazon. Although our cross-sectional design has no power to prove causality, we believe that understanding the possible health impacts of mercury exposure among the Munduruku children is vital, not only for developing strategies to mitigate these effects but also for supporting the Munduruku people’s fight for their rights and the protection of their territories. The findings of this study will serve as a foundation for advocating for more effective regulatory measures, increased resources for monitoring, and initiatives aimed at reducing exposure and preventing further contamination in these vulnerable communities.
